# Unsteady Boundary-Layer Flow over Jerked Plate Moving in a Free Stream of Viscoelastic Fluid

**DOI:** 10.1155/2014/601950

**Published:** 2014-05-06

**Authors:** Sufian Munawar, Ahmer Mehmood, Asif Ali, Najma Saleem

**Affiliations:** ^1^Department of Mathematics and Basic Sciences, Prince Mohammad Bin Fahd University, Al Khobar 31952, Saudi Arabia; ^2^Department of Informatics and Systems, University of Management and Technology, Lahore 54000, Pakistan; ^3^Department of Mathematics (FBAS), International Islamic University Islamabad, Islamabad 44000, Pakistan; ^4^Department of Mathematics, Quaid-i-Azam University, Islamabad 44000, Pakistan

## Abstract

This study aims to investigate the unsteady boundary-layer flow of a viscoelastic non-Newtonian fluid over a flat surface. The plate is suddenly jerked to move with uniform velocity in a uniform stream of non-Newtonian fluid. Purely analytic solution to governing nonlinear equation is obtained. The solution is highly accurate and valid for all values of the dimensionless time 0 ≤ *τ* < *∞*. Flow properties of the viscoelastic fluid are discussed through graphs.

## 1. Introduction

Boundary-layer flows over flat surfaces are fundamental for the understanding of aerodynamical properties of the flow such as wall friction and the dynamical drag. Due to the boundary-layer theory proposed by Prandtl in 1904 [1], it became possible to calculate the drag on a sphere accurately. Blasius [2] investigated the boundary-layer flow past a flat plate at zero incidence. The wall friction in Blasius flow was calculated to be 0.332057 approximately. The same two-dimensional boundary-layer flow was studied by Sakiadis [3] over a moving wall in a still fluid. Sakiadis reported the value of skin friction at the wall to be 0.444… for his case. Sakiadis also predicted that the drag force for his flow problem was 34% greater than the drag force for the Blasius flow [2]. The theoretical results of Sakiadis [3] were experimentally confirmed by Tsuo et al. [4]. In both studies [2, 3], either fluid or plate is assumed to be at rest. However, the most practical situation might be the one in which both the fluid and the plate are moving. Such a flow situation is more practical in studying the aerodynamical properties of the flow. Keeping this fact in mind, Klemp and Acrivos [5] considered two-dimensional boundary-layer flow of a viscous fluid over a flat surface moving in a stream of constant velocity. Hussaini et al. [6] determined similarity solution of the boundary layer equation with upstream moving wall. Later on in 2003, Fang [7, 8] studied the similarity solution of boundary-layer flow and heat transfer for steady case. In [7, 8], Fang studied both cases, namely, when the plate is moving in the direction of free stream or in opposite direction to the free stream. Recently, Mehmood et al. [9, 10] considered unsteady boundary-layer flow over an impulsively started moving plate in a free stream with parallel and antiparallel motion.

Boundary-layer flows over moving surfaces find important industrial applications such as in the manufacturing of food and paper, plastic sheet extrusion, application of coating of paints layers on surfaces, and many other activities (see for instance [11, 12]). In such industrial applications, the fluid is observed to have non-Newtonian character. Due to these important industrial applications, the study of non-Newtonian fluid flows over moving surface needs attention. In 1969, Fox et al. [13] investigated the non-Newtonian flow over a moving surface using the power-law model. The flow of a power-law fluid over a moving plate in a parallel free stream was investigated by Hassanien [14]. Further, Hassanien [15] investigated the boundary-layer flow over a moving plate in a free stream of second-grade fluid with heat transfer analysis.

In all the abovementioned studies for non-Newtonian fluids, the authors considered steady flow. However, there are many practical applications in which the flow is essentially unsteady. For unsteady problems governed by nonlinear equations, it is very difficult to obtain an analytic solution valid for all time. Perturbation methods are sometimes used to get analytic solution valid for small time [16–18]. Currently, Liao [19, 20], Xu et al. [21], Cheng et al. [22], Wang [23], Abbasbandy et al. [24], Xu and Liao [25], Mehmood et al. [9, 10, 26, 27], and Munawar et al. [28] obtained purely analytic solution for unsteady flows by homotopy analysis method [29]. In [9, 10, 19–28], the authors have shown their results to be valid uniformly for all time. Homotopy analysis method is a powerful analytic technique (introduced by Liao [29]) for highly nonlinear problems. The technique has been widely used by a number of researchers in nonlinear problems arising in science and engineering [30–41].

In the present study, we extend the work of Mehmood and Ali [9] for the non-Newtonian case. The fluid considered is of second-grade type and the problem is solved by homotopy analysis method. The objective of this study is twofold: firstly to report a purely analytic solution to the considered unsteady problem and secondly to investigate the viscoelastic effects on the flow characteristics. The solution is highly accurate and is uniformly valid for all time in the whole spatial domain. The accuracy and convergence of present analytic solution are discussed in detail. The outlines of the paper are as follows.

Section 2 contains the mathematical formulation and the HAM solution of the problem. The issue of convergence and accuracy of HAM results is also discussed in Section 2.  Section 3 consists of graphical representation of results and their discussion.  Section 4 is reserved for concluding remarks.

## 2. Formulation of the Problem

Consider the unsteady incompressible flow of second-grade fluid over an infinite plate at *y* = 0. The fluid occupies the half space *y* > 0 and at infinity it is assumed that fluid is flowing with the constant free-stream velocity *U*. Initially, fluid is at rest; then at time *t* ≥ 0, suddenly plate starts its motion with the constant velocity *λU*, where *λ* is the ratio of plate velocity to the free-stream velocity. For an incompressible homogeneous fluid of second-grade type, the Cauchy stress tensor **T** is related to the deformation field through

(1) $$\begin{array}{c} T=-pI+\mu A_{1}+\alpha_{1}A_{2}+\alpha_{2}A_{1}^{2}, \end{array}$$

where *p* is the pressure, **I** is the identity tensor, *μ* is the dynamic viscosity, *α*_1_ and *α*_2_ are the viscoelastic parameters, and the kinematical tensors **A**_1_ and **A**_2_ are given by

(2) $$\begin{array}{c} A_{1}=L+L^{T},\\ A_{2}=\frac{dA_{1}}{dt}+A_{1}L+L^{T}A_{1}, \end{array}$$

where *d*/*dt* is the material time derivative and **L** is velocity gradient. This rheological model was first introduced by Rivlin and Ericksen [42].

Experimental data available for the large number of viscoelastic fluids suggests that, in order to satisfy the thermodynamical analysis, some restrictions must be put on the signs and magnitudes of the material parameters [43]

(3) $$\begin{array}{c} \mu \geq 0,\;\;\alpha_{1}\geq 0,\;\alpha_{1}+\alpha_{2}=0. \end{array}$$

Under the above assumptions and conditions, the boundary layer equations governing the unsteady laminar flow of an incompressible viscoelastic fluid due to an impulsive motion of plate are given by

(4) $$\begin{array}{c} \frac{\partial u}{\partial x}+\frac{\partial v}{\partial y}=0, \end{array}$$

(5) $$\begin{array}{c} \frac{\partial u}{\partial t}+u\frac{\partial u}{\partial x}+v\frac{\partial u}{\partial y}=\frac{\partial T_{xx}}{\partial x}+\frac{\partial T_{xy}}{\partial x}, \end{array}$$

where *T*_*xx*_ and *T*_*xy*_ are the components of the stress tensor given by

(6) Txx=−p+2μ∂u∂x+α1[2∂2u∂t∂y+2u∂2u∂x2+2v∂2u∂x∂y+(∂v∂x)2−(∂u∂y)2],

(7) Txy=μ(∂u∂y)+α1[∂2u∂t∂y+u∂2u∂x∂y+v∂2u∂y2+2∂u∂x∂u∂y].

Using (6) and (7) in (5) and assuming the constant pressure, we have

(8) ∂u∂t+u∂u∂x+v∂u∂y =ν∂2u∂y2+α1ρ[∂2u∂t∂y2+∂∂x(u∂2u∂y2)+v∂3v∂y3+∂u∂y∂2v∂y2],

subject to the boundary conditions (when *t* ≥ 0)

(9) u=λU, v=0, at  y=0,u⟶U, as  y⟶∞,

in which *ν* is the kinematic viscosity, *ρ* is the fluid density, and *u*(*x*, *y*, *t*) and *v*(*x*, *y*, *t*) are the velocity components in the *x*- and *y*-directions, respectively. The initial condition (when *t* < 0) is given by

(10) $$\begin{array}{c} u=v=0,\;\forall x,y. \end{array}$$

Introducing the similarity transformations [9]

(11) η=Uνxξy,  ψ=νUxξf(η,ξ),ξ=1−e−τ,  τ=Uxt,

the governing equation (8) readily transforms to

(12) [ξ−α(1−ξ)]f′′′−ξ2(1−ξ)∂f′∂ξ+12ηξ(1−ξ)f′′  +12ξ2ff′′=−α[ξ(1−ξ)∂f′′′∂ξ−η2(1−ξ)fiv  +12ξf′′2−12ξffiv−ξf′f′′′],

with boundary conditions

(13) $$\begin{array}{c} f\left(0,\xi \right)=0,\;{\frac{\partial f}{\partial \eta }|}_{\eta =0}=\lambda ,\;{\frac{\partial f}{\partial \eta }|}_{n\to \infty }=1, \end{array}$$

where *f*(*η*, *ξ*) is the dimensionless stream function, *α* = *α*_1_ *U*/*ρxν* is the local Deborah number, and ′ denotes differentiation with respect to *η*. Notice that, for *α* = 0, the above equation reduces to that of the viscous fluid [9].

## 3. HAM Solution

To solve the problem (12) and (13) analytically, we use the well-known analytic technique homotopy analysis method. According to boundary conditions (13), it is convenient to express *f*(*η*, *ξ*) by the base functions

(14) $$\begin{array}{c} \left\{\xi^{k}\eta^{m}\exp\left(-n\eta \right)\;|\;k\geq 0,\;\;m\geq 0,\;\;n\geq 0\right\} \end{array}$$

in the following form:

(15) $$\begin{array}{c} f\left(\eta ,\xi \right)=\overset{\infty }{\underset{k=0}{\sum }}\;‍\overset{\infty }{\underset{m=0}{\sum }}‍\;\overset{\infty }{\underset{n=0}{\sum }}a_{m,n}^{k}\xi^{k}\eta^{m}\exp\left(-n\eta \right), \end{array}$$

where *a*_*m*,*n*_^*k*^ are coefficients involved in the solution series. According to the solution expression (15) and boundary conditions (13), we choose the initial approximation

(16) $$\begin{array}{c} f_{0}\left(\eta ,\xi \right)=\eta -\frac{1-\exp\left(-\beta \eta \right)}{\beta }+\lambda \eta \exp\left(-\beta \eta \right) \end{array}$$

and the linear operator

(17) $$\begin{array}{c} L\left[F\left(\eta ,\xi ;p\right)\right]=\frac{\partial^{3}F}{\partial \eta^{3}}+\beta \frac{\partial^{2}F}{\partial \eta^{2}} \end{array}$$

satisfying the property

(18) $$\begin{array}{c} L\left[C_{1}+C_{2}\eta +C_{3}\exp\left(-\beta \eta \right)\right]=0, \end{array}$$

where *C*_1_, *C*_2_, and *C*_3_ are constants and *β* is the controlling parameter to be adjusted. From (12), we define the nonlinear operator

(19) N[F(η,ξ;p)] =[ξ−α(1−ξ)]F′′′(η,ξ;p)−ξ2(1−ξ)∂F′(η,ξ;p)∂ξ  +12ηξ(1−ξ)F′′(η,ξ;p)+12ξ2F(η,ξ;p)F′′(η,ξ;p)  +α[ξ(1−ξ)∂F′′′(η,ξ;p)∂ξ−η2(1−ξ)Fiv(η,ξ;p)  +ξ2F′′2(η,ξ;p)−ξF′(η,ξ;p)F′′′(η,ξ;p)  −ξ2F(η,ξ;p)Fiv(η,ξ;p)].

Taking *ℏ* as nonzero auxiliary parameter, we construct the zero-order deformation equation [29]

(20) $$\begin{array}{c} \left(1-p\right)L\left[F\left(\eta ,\xi ;p\right)-f_{0}\left(\eta \right)\right]=p\hbar N\left[F\left(\eta ,\xi ;p\right)\right], \end{array}$$

subject to the boundary conditions

(21) F(0,ξ;p)=0, ∂F(η,ξ;p)∂η|η=0=λ,  ∂F(η,ξ;p)∂η|η→∞=1,

where *p* is the embedding parameter and obviously, when *p* = 0 and *p* = 1, (20) has the solutions

(22) F(η,ξ;0)=f0(η,ξ),

(23) F(η,ξ;1)=f(η,ξ),

respectively. Thus, the variation of *p* from 0 to 1 is continuous deformation of *f*(*η*, *ξ*) from *f*_0_(*η*, *ξ*) to *f*(*η*, *ξ*). Expanding *F*(*η*, *ξ*; *p*) in Taylor's series with respect to *p*, and using (22), we have

(24) $$\begin{array}{c} F\left(\eta ,\xi ;p\right)=f_{0}\left(\eta ,\xi \right)+\overset{\infty }{\underset{m=1}{\sum }}f_{m}\left(\eta ,\xi \right)p^{m}, \end{array}$$

where

(25) $$\begin{array}{c} f_{m}\left(\eta ,\xi \right)={\frac{1}{m!}\frac{\partial^{m}F\left(\eta ,\xi ;p\right)}{\partial p^{m}}|}_{p=0}. \end{array}$$

Assume that *ℏ* is chosen so properly such that the series (24) is convergent at *p* = 1. Using (22) and (23), we can write

(26) $$\begin{array}{c} f\left(\eta ,\xi \right)=f_{0}\left(\eta ,\xi \right)+\overset{\infty }{\underset{m=1}{\sum }}f_{m}\left(\eta ,\xi \right). \end{array}$$

Differentiating the zero-order deformation equations (20) and (21) *m*-times with respect to *p*, at *p* = 0 and then dividing by *m*!, we obtain the *m*th*-*order deformation equation

(27) $$\begin{array}{c} L\left[f_{m}\left(\eta ,\xi \right)-\chi_{m}f_{m-1}\left(\eta ,\xi \right)\right]=\hbar R_{m}\left(\eta ,\xi \right) \end{array}$$

subject to the boundary conditions

(28) $$\begin{array}{c} f_{m}\left(0,\xi \right)=0,\;{\frac{\partial f_{m}}{\partial \eta }|}_{\eta =0}={\frac{\partial f_{m}}{\partial \eta }|}_{\eta \to \infty }=0, \end{array}$$

where

(29) Rm(η,ξ) =[ξ−α(1−ξ)]fm−1′′′−α2η(1−ξ)fm−1iv  +12ηξ(1−ξ)fm−1′′+αξ(1−ξ)∂fm−1′′′∂ξ  −ξ2(1−ξ)∂fm−1′∂ξ+12ξ2∑k=0m−1fm−1−kfk′′  +α[−ξ∑k=0m−1fm−1−k′fk′′′−12ξ∑k=0m−1fm−1−kfkiv+12ξ∑k=0m−1fm−1−k′′fk′′],χm={0,m=11,m>1.

The system of linear nonhomogeneous equations (27)-(28) can be solved up to high order of approximation with the help of the symbolic computation software such as* Mathematica*.

Let *f*_m_*(*η*, *ξ*) represent a special solution; then the general solution *f*_*m*_(*η*, *ξ*) can be determined as follows:

(30) $$\begin{array}{c} f_{m}\left(\eta ,\xi \right)=f_{m}^{*}\left(\eta ,\xi \right)+C_{1}+C_{2}\eta +C_{3}e^{-\beta \eta }, \end{array}$$

in which *C*_*i*_  (*i* = 1,…, 3) are constants of integration which can be determined with the help of boundary conditions (28) as under

(31) $$\begin{array}{c} C_{1}=-C_{3}-f_{m}^{*}\left(0,\xi \right),\;\;\;C_{2}=0,\;\;C_{3}=-\frac{1}{\beta }f_{m}^{*\prime }\left(0,\xi \right). \end{array}$$

In this way, the complete solution for the present problem can be written in the form of an infinite series of functions; that is,

(32) $$\begin{array}{c} f\left(\eta ,\xi \right)=f_{0}\left(\eta ,\xi \right)+\overset{\infty }{\underset{m=1}{\sum }}f_{m}\left(\eta ,\xi \right). \end{array}$$

**Figure 1:**
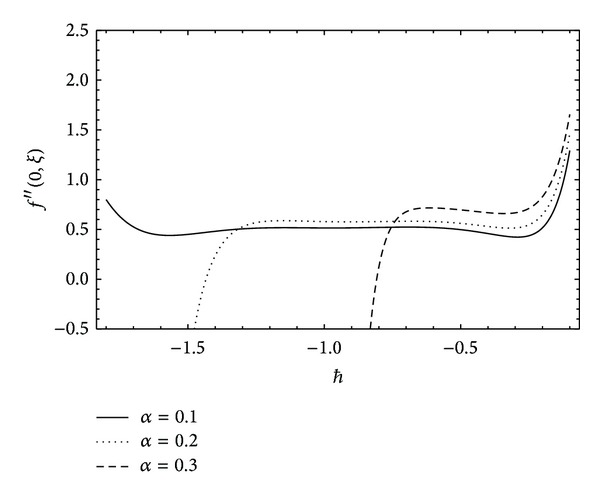
*ℏ*-Curve for different values of the Deborah number *α* at the 19th order of approximation.

**Figure 2:**
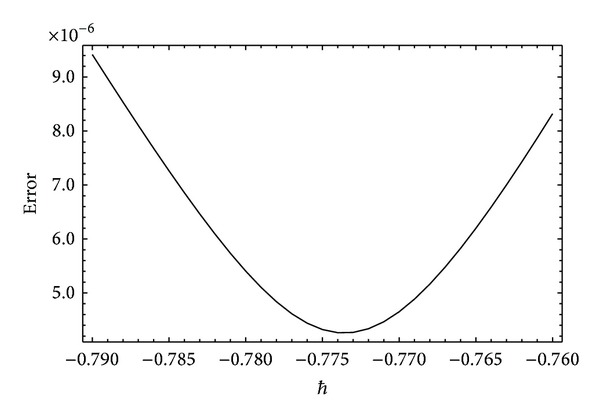
Squared relative error for the optimal values of *ℏ* for *α* = 0.1 by keeping *ξ* = 0.8, *λ* = 0, and *β* = 5 fixed.

**Figure 3:**
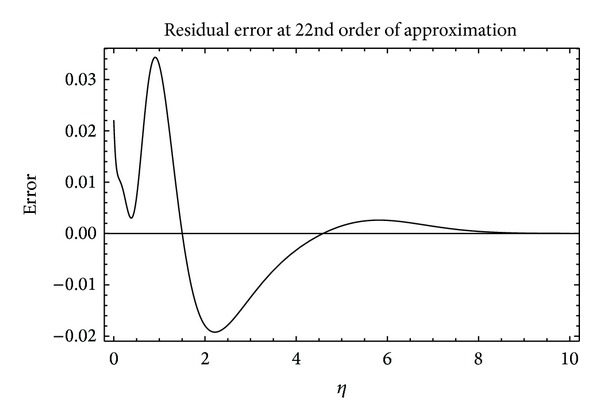
The residual error of the 22nd order of HAM solution in case of *α* = 0.2, *ξ* = 0.8, *λ* = 0, *β* = 5, and *ℏ* = −0.6815.

To prove that the series *f*(*η*, *ξ*) is an approximate solution of the system (12) and (13), it is necessary to show the convergence of the solution series (32). As mentioned by Liao [29], the convergence of the solution series strongly depends upon the auxiliary parameter *ℏ* once the initial guess and the linear operator have been selected. A rough estimate for the allowed regions of the values of *ℏ*, the so-called *ℏ*-curve, is important. We have plotted the *ℏ*-curve for our present problem in Figure 1. It is worth mentioning here that, in some problems with strong nonlinearity, the values of *ℏ* strongly depend upon the involved physical parameters (see for instance [44]). In Figure 1, it can be seen that the intervals of allowed values of *ℏ* are shrinking with increasing *α*. Further, it can also be seen that, for higher values of *α*, the interval of allowed values of *ℏ* shifts towards zero. However, to search a more appropriate value of *ℏ*, it is useful to calculate the residual errors. In Figure 2, we have plotted the residual error against *ℏ* for fixed values of the parameters involved. From Figure 2, it is clear that the error for *α* = 0.1 is minimum at *ℏ* = −0.7736 which is 4.257 × 10^−6^ at the 20th order of approximation. In Figure 3, we have plotted the errors graph against the space variable *η* also. It shows that in the boundary layer region the error fluctuates and dies out to be zero as one moves to the outer region. Though the error fluctuates in the boundary layer region, it remains in the acceptable limits. In order to prove the convergence of the solution series, it is recommended that the corrections to solution must become negligible in the succeeding orders of approximation. We apply the homotopy Padé approximation in order to accelerate the convergence of solution series. In Table 1, we report the HAM solution at different orders of [*m*, *m*] Padé approximation. Clearly, there are no corrections up to four decimal places after the 8th order of Padé approximation. This proves the convergence and accuracy of HAM solution.

## 4. Graphical Illustration and Discussion of Results

**Figure 4:**
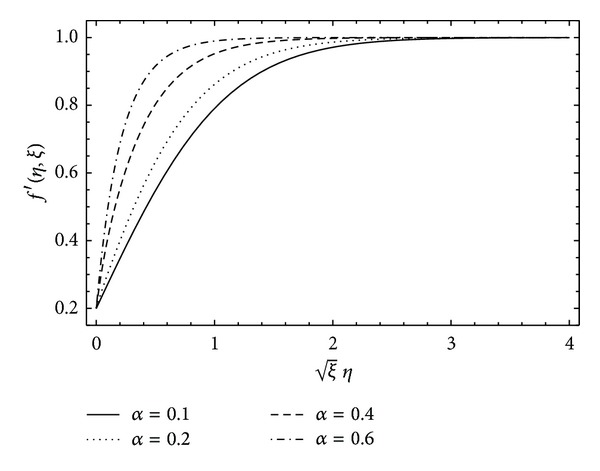
Effect of viscoelastic parameter *α* on the velocity profile when *λ* = 0.2, *ξ* = 0.5, and *β* = 5 are kept fixed.

**Figure 5:**
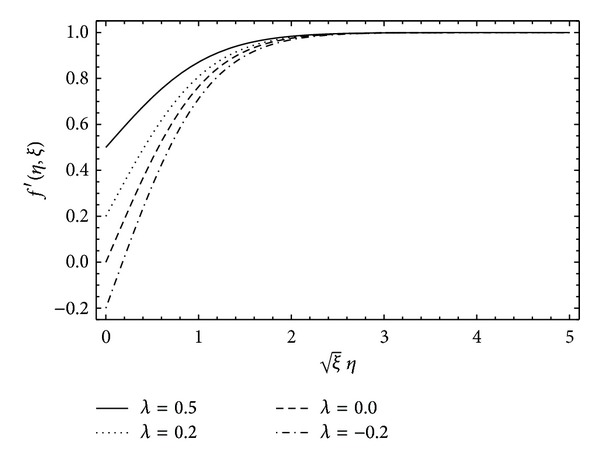
Effect of the parameter *λ* on the velocity profile when *α* = 0.1, *ξ* = 0.5, and *β* = 5 are kept fixed.

**Figure 6:**
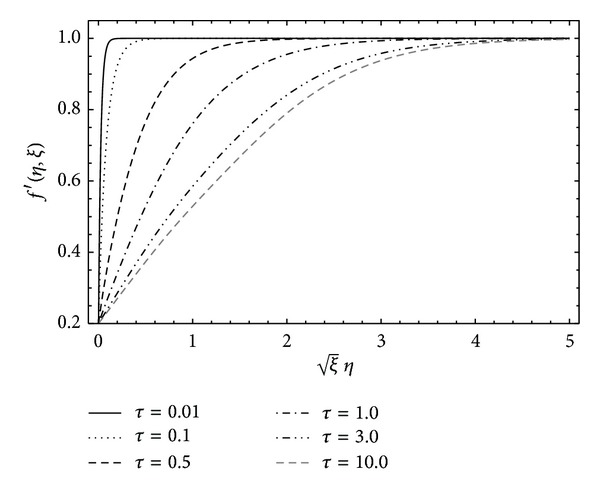
Velocity profile for different time *τ* when *α* = 0.2, *λ* = 0.2, and *β* = 5 are kept fixed.

To understand the physics of flow phenomenon, we have investigated the solution through graphs. In Figure 4, the longitudinal component of velocity *f*′(*η*, *ξ*) is plotted for different values of the viscoelastic parameter *α*. Clearly, strong non-Newtonian behavior of the fluid results in large skin friction at the solid wall. However, large values of *α* help in reducing the thickness of the boundary layer. In Figure 5, the velocity function *f*′(*η*, *ξ*) is plotted for different values of the parameter *λ*  (provided *λ* ∈ (0,1)). The velocity increases at the plate which causes the skin friction at the plate to reduce. This is due to the fact that, for *λ* > 0, the plate and the free stream progress in the same direction, but, for *λ* < 0 when the plate and free stream have opposite direction of progression, the skin friction increases considerably. Finally, the present HAM solution is shown to be uniformly valid for all time 0 ≤ *τ* < *∞* in the whole spatial domain 0 ≤ *η* < *∞* in Figure 6. Figure 6 depicts that the velocity is very small at initial time and with the passage of time the flow develops and reaches its steady state due to the motion of the plate. From the figure, it can also be observed that the steady state is reached at *τ* = 5 (roughly). This shows that the vorticity diffusion takes place in a very short interval of time.

The effect of viscoelastic parameter *α* on the boundary layer thickness *δ* is shown in Table 2. It is observed from the table that the thickness of the boundary layer, *δ*, has a declining behavior with an increase in *α*. For the low value of *α*, this drop in *δ* is less as compared with the high value of *α*. Such kind of result can be expected because at low values of *α* our governing equation behaves like that of Newtonian fluid [45]. At the low values of *α*, shear stress produced in the flow is not so large so the boundary layer thickness increases. By increasing *α*, the shear stress will grow more and more and results in the reduction of boundary layer thickness. We can observe such kind of results from the theory of polymeric liquid as well as experimental observations.

## 5. Concluding Remarks

In this study, we have considered the unsteady boundary-layer flow of a viscoelastic fluid over an impulsively started moving flat plate. Fluid at infinity was assumed to be flowing with a uniform free stream velocity. The governing nonlinear equations of an incompressible second-grade fluid are modelled using the similarity transformations. The resulting nonlinear problem is solved analytically with the help of HAM. The influence of various parameters of interest on the velocity profile is graphically illustrated. We find the following observations.

- It is noticed that the boundary layer thickness *δ* decreases by increasing second-grade parameter *α*.
- It is observed that as the time passes by, the unsteady velocity reaches the steady state showing that our analytic solution is valid for all the time throughout the spatial domain.
- It is observed that increasing *λ* (for *λ* ∈ (0,1)) reduces the drag at the plate whereas for *λ* < 0 the skin friction at the plate increases by increasing |*λ*|.

## Figures and Tables

**Table 1:** Convergence of HAM solution for different orders of padé approximation of *f*′′(0, *ξ*), when *ξ* = 0.8, *α* = 0.2, *β* = 5 and *λ* = 0.

Order of approximation	*f*′′(0, *ξ*)
[2,2]	0.4201
[4,4]	0.5460
[6,6]	0.5526
[8,8]	0.5650
[10,10]	0.5650
[12,12]	0.5650

**Table 2:** Effect of viscoelastic parameter *α* on the boundary layer thickness *δ*, when *λ* = 0.2, *β* = 5 and *ξ* = 0.5.

*α*	Boundary layer thickness *δ*
0.00	2.2221
0.01	2.2030
0.05	2.3375
0.10	2.4656
0.20	2.1302
0.40	1.5352
0.60	1.0112
0.80	0.9339
1.00	0.8981
1.20	0.8464
